# Long-Term AVF Patency - Can we do better?

**DOI:** 10.1590/2175-8239-JBN-2019-0063

**Published:** 2019-06-27

**Authors:** Tushar J. Vachharajani, Georges Nakhoul, Jonathan J. Taliercio

**Affiliations:** 1 Cleveland Clinic Foundation Cleveland, Glickman Urological & Kidney Institute Department of Nephrology & Hypertension ClevelandOH USA Cleveland Clinic Foundation, Cleveland, Glickman Urological & Kidney Institute, Department of Nephrology & Hypertension, Cleveland, OH, USA.

Hemodialysis (HD) remains a principal modality for renal replacement therapy worldwide for patients with end stage kidney disease (ESKD) and a functioning dialysis vascular access is critical to providing adequate therapy. Despite all the technological advances in the delivery of HD, the process of creating a vascular access has remained vastly unchanged. Almost six decades since the creation of an autogenous fistula, the dialysis community continues to struggle with creating a dependable method to remove blood from the body for HD therapy^1^. The three most commonly used dialysis vascular access types continue to be native arteriovenous fistula (AVF), arteriovenous graft using a synthetic material (AVG) and a central venous catheter (CVC). Each of these vascular access types has its own advantages and disadvantages. However, the general consensus guidelines from multiple professional societies recommend AVF as the most preferred access type over AVG and CVC primarily because of lower mortality, cost, infection rate, incidence of thrombosis, fewer interventions, and longer patency^2^. The major challenges with creating a functional AVF have been already identified since the implementation of the Fistula First Breakthrough Initiative (FFBI) in the United States in the mid 1990’s (List).^3^

List: Barriers for timely creation of an arteriovenous fistula

1. System-related barriers:
  - Late referral to a nephrologist by primary care physicians;
  - Late referral to surgeons for AV access creation by nephrologists;
  - Backlog with surgeons - clinic and operating room appointments;
  - Lack of defined process for follow up visits after the surgery;
  - Poor understanding of timely intervention for a primary non-maturing AVF.
2. Patient-related barriers:
  - Denial phase, non-compliance;
  - Lack of education;
  - Aging population;
  - Multiple comorbidities;
  - Socio-economic factors, such as transportation, family support, co-payments etc.
3. Variable provider expertise:
  - Inadequately trained nephrologists, interventionalists and surgeons;
  - Minimal importance to vascular access in training curricula;
  - Inadequate emphasis on vessel preservation in advanced CKD population amongst all healthcare providers;
  - Inadequately trained allied health providers;
  - Limited training resources.
4. III-defined barriers:
  - Poorly understood pathophysiology of AVF dysfunction;
  - Hemodynamic impact of AVF.

Siga E et al. have focused on the importance of a multidisciplinary access team inclusive of a dedicated surgeon towards creating a successful AVF^4^. The primary unassisted and functional primary patency rates in their series of 113 AVF created by a single surgeon and same support team over a 12-year period was 70.6% and 80.9% respectively. The authors relate the long-term benefits to a multidisciplinary team approach that is consistent and organized. Unfortunately, larger scale studies have not been able to duplicate their results. The challenge of duplicating such results, especially with consistent long-term AVF patency on a larger scale remains unsolved and elusive.

Many lessons have been learned over the past two decades since the implementation of FFBI and National Kidney Foundation-Kidney Disease Outcomes Quality Initiatives (NKF-KDOQI) Vascular Access Guidelines that can guide our future management goals.

The first objective should focus on building a multi-disciplinary team consisting of a lead nephrologist, a skilled and dedicated surgeon, an interventionalist, engaged dialysis staff, a vascular access coordinator, and pre-dialysis counselor to help motivate and educate the patient. When possible, vascular access-focused education and planning should begin early enough to avoid/minimize the need to use CVC. KDIGO 2006 guidelines recommend that AVF should be placed at least 6 months before the anticipated start of HD treatments. Some authors have proposed placement of AVF at GFR ≤20 mL/min/1.73 m^2^.^5^ Finally, preservation of artery and veins is recommended, by minimizing venipuncture or other centrally inserted lines in CKD 4-5 patients in order to maximize the creation of a distal upper extremity AVF.^6^

The second objective should focus on identifying a non-maturing AVF within six to eight weeks of placement in order to plan for early intervention and assist with the maturation process. The ultimate goal being minimizing the CVC exposure time and preserving the central veins. This goal can be achieved if the caregivers and patients are trained with proper physical examination technique to identify a problematic AVF. In general, mature AVF should have an access flow of 600 mL/min, be less than 0.6 cm below the surface of the skin, and have the minimal diameter of 0.6 cm (Rule of 6s). The FFBI team has created several tools that can help improve the competency skills of the entire dialysis community^7^.

The last objective should focus on strategies to improve long-term patency of a functioning AVF. Several hurdles have been identified in achieving this goal and the biggest barrier of all is breaking “old habits”. Long-term AVF patency can be improved if collectively, as dialysis caregivers, we implement a fundamental change in our practice patterns. All well-functioning AVFs invariably become dysfunctional over time. Long-term patency of AVFs can be achieved by improving the overall process of care. Several key steps must be taken to ensure the functionality of the access over an extended period of time. These steps include:

- Assessment and proper cannulation technique - Inadequately trained dialysis personnel can cause major trauma to the vessel, often irreversible. Frequent staff training and competency assessment for aseptic technique, correct needle size, proper cannulation technique, and needle anchoring procedure are fundamental for maintaining patency^8^^,^^9^.
- Early identification of dysfunction - History and physical examination to assess the presence of stenosis, thrombosis, aneurysms, and recognizing prolonged bleeding times and access pressures, which should be recognized before and after each treatment for timely intervention. Using monitoring and surveillance data, such as ultrasound, access blood flow and pressure, are complimentary evidence to identify dysfunctional AVFs. Timely recognition of dysfunction can lead to timely referral for intervention.
- Identifying complications - Recognizing signs of infection, monitoring for aneurysms, symptoms of distal limb ischemia, and impact on cardiac hemodynamics are relevant for providing optimal care^10^.
- Empowering the patient - Every attempt should be made to educate and empower the patients to participate in their care. Engaged and involved patients can identify certain problems before the complication becomes a major issue. An engaged patient may be able to identify a change in the character of the thrill within an AVF before a thrombotic event. Success from early endovascular intervention in a poorly flowing AVF is higher than in a completely thrombosed access^11^.
- Training the next generation - Nephrology curriculums across the globe place little emphasis on dialysis vascular access. Lessons learned from the current generation will improve the quality of care for the next generation of patients. The worldwide epidemic of ESKD and the economic impact of dialysis therapy on society warrants a major shift in the training curriculum for all caregivers. Innovative research and better understanding of the changes in vascular biology following AVF creation may offer better options for care in the future.
